# Kindlin1 As a Gender and Location-Specific Diagnostic Marker in Gastric Cancer Patients

**DOI:** 10.30699/IJP.2021.526950.2603

**Published:** 2021-12-15

**Authors:** Mohammad Reza Abbaszadegan, Negin Taghehchian, Azadeh Aarabi, Sohrab Nozari, Ehsan Saburi, Meysam Moghbeli

**Affiliations:** 1 *Medical Genetics Research Center, Mashhad University of Medical Sciences, Mashhad, Iran*; 2 *Department of Chemistry, Faculty of Science, Ferdowsi University of Mashhad, Mashhad, Iran*; 3 *Immunology Research Center, Mashhad University of Medical Sciences, Mashhad, Iran*; 4 *Student Research Committee, Faculty of Medicine, Mashhad University of Medical Sciences, Mashhad, Iran*; 5 *Department of Medical Genetics and Molecular Medicine, School of Medicine, Mashhad University of Medical Sciences, Mashhad, Iran*

**Keywords:** Cell adhesion, Extracellular matrix, Gastric cancer, Helicobacter Pylori, Integrin

## Abstract

**Background & Objective:**

Gastric cancer (GC) is considered one of the main reasons for cancer-related mortalities among Iranians. Kindlin-1 is an adhesion protein member of integrin-interacting proteins, regulating integrin activation through direct interaction with β-integrin. Therefore, kindlin-1 can be involved in the regulation of cell proliferation and adhesion. In the present study, we assessed the possible role of kindlin-1 in GC progression and metastasis.

**Methods:**

KINDLIN1 messenger RNA (mRNA) expression was assessed in tumor tissues from 80 GC patients in comparison with normal tissues using real-time polymerase chain reaction (PCR).

**Results:**

The levels of KINDLIN1 expressions were significantly correlated with sex (*P*=0.05) and tumor location (*P*=0.002). KINDLIN1 expression was also significantly associated with lymph node metastasis among the *Helicobacter Pylori* (HP)-negative cases (*P*=0.001). Moreover, a significant association between age and KINDLIN1 expression was observed among the HP-positive cases (*P*=0.039).

**Conclusion:**

In the present study, we introduced KINDLIN1 as a location-specific marker for cardia gastric carcinoma. Moreover, it was observed that KINDLIN1 could be used as a gender-dependent diagnostic marker of GC patients.

## Introduction

Gastric cancer (GC) is considered the second leading reason of cancer-related mortalities globally (1). Genetic and environmental factors are involved in the initiation and progression of GC. Therefore, introducing novel, sensitive biomarkers is desired to clarify the biology of GC and develop new targeted therapies. Although early-stage GC patients have a five-year survival rate of up to 95%, the survival rate of advanced-stage cases significantly decreases. Kindlin-1 is an adhesion protein member of the kindlin family (2). These integrin-interacting proteins regulate integrin activation through direct interaction with β-integrin. Therefore, kindlin-1 is involved in regulating cell proliferation and adhesion (3, 4). 

Kindlin-1 expression is reported in many tumors, including lung, breast, and colon (5, 6). Tumor metastasis is a multistep process in which tumor cells invade the margins, blood circulation, extravasation from blood vessels, and micrometastases in distant secondary tissues. Every step needs molecular alterations of specific metastatic genes (7). Moreover, it has been reported that kindlin-2 activates Wingless and Int-1 (Wnt) signaling via β-catenin stabilization (8). Kindlin-1 interacts with transforming/tumor growth factor β (TGF-β)/ SMAD family member 3 (Smad3) signaling components to promote the TGF-β-induced migration in colorectal cancer (9). It is required to introduce novel early detection markers to improve therapeutic methods. To our knowledge, no study has reported kindlin-1 expression in GC. Therefore, in the present study, we assessed the possible role of kindlin-1 in GC progression and metastasis.

## Material and Methods

**Tissue Samples**

Fresh tumor and normal gastric tissues were obtained through gastric surgery at Emam Reza and Omid Hospitals of Mashhad University of Medical Sciences, Mashhad, Iran. Eighty patients were enrolled who did not receive any chemo- or radio-therapeutic treatments. Specimens were immediately kept in RNA later solution and stocked at -20°C until extraction. All participants signed informed consent forms. 

**RNA Extraction and Comparative Real-Time Polymerase Chain Reaction**

Total RNA was extracted from frozen GC and normal tissues using the RNeasy Mini Kit (Qiagen, Hilden, Germany). Complementary DNA (cDNA) synthesis was performed by a cDNA synthesis kit (Parstous, Iran). Comparative real-time polymerase chain reaction (PCR) was performed using the LightCycler 96 instrument (Roche, Germany). The expression level of kindlin-1 was analyzed using the SYBR Green method (ParsTous, Iran); primer sequences are presented in Table 1. Data were normalized using glyceraldehyde 3-phosphate dehydrogenase (GAPDH). The fold changes bigger than +2 and lower than -2 were considered over- and underexpressions, respectively.

**Table 1:** Primer sequences used for quantitative real-time RT-PCR

Name	Forward primer sequence	Reverse primer sequence
KINDLIN-1	5- GTTGGAGGAGTGATGCTCAAGTTAG -3	**5- ATTTTATGCTGAGGGGTGAAGAGA -3**
GAPDH	5-GGAAGGTGAAGGTCGGAGTCA-3	**5-GTCATTGATGGCAACAATATCCACT-3**

**Statistical Analysis**

All statistical analyses were performed using SPSS 19.0 (SPSS Inc., Chicago, Ill., USA). The associations between gene expressions and clinicopathological factors were evaluated by the *v*2 test or Fisher’s exact test (*P*≤0.05)**.**

## Results

**Study Population and KINDLIN1 Messenger RNA Expression**

**Fig. 1:**
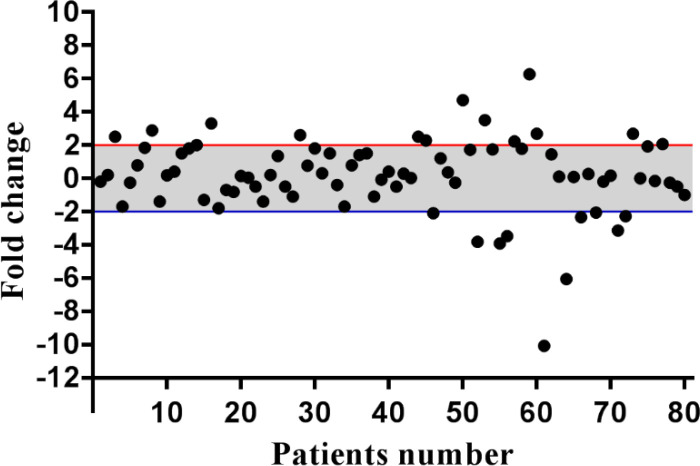
Descriptive analysis of relative gene expression of KINDLIN1 in GC patients. The thresholds for the over and under-expressed cases are shown by the red and blue lines, respectively. The grey area indicates the cases with normal level of KINDLIN1 mRNA expression

Eighty GC patients were enrolled in the present study. The numbers of tumors in non-cardia were higher compared to the tumors located in the cardia (47/80 vs. 33/80). Metastatic lymph nodes were also observed in 67 out of 80 cases (83.8%). Forty-nine out of 80 tumors (61.2%) were moderately differentiated. *Helicobacter Pylori* (HP) was observed in 36 out of 68 cases (45%) who had HP results. Demographic and clinicopathological features are presented in Table 2. KINDLIN1 messenger RNA (mRNA) expression was assessed in 80 tumor tissues in comparison with normal margins. Fourteen (17.5%) and 10 (12.5%) out of 80 cases had Knidlin-1 over- and underexpression, respectively. KINDLIN1 fold changes were ranged from -10.06 to 6.27 fold changes (mean fold±SD, 0.17±2.28; Figure 1). The mean fold changes for the over-and underexpressed tissues were 3.02±0.31 (mean±SD) and -3.92±0.78 (mean±SD), respectively.

**Table 2:** Correlation between the level of KINDLIN1 mRNA expression and clinicopathological features of GC patients

	Total	KINDLIN1 over expression	KINDLIN1 under expression	P-value
Patients	80	14(17.5%)	10(12.5%)	
Mean age (Years, mean ± SD)	63.08±11.23	61.07±1.92	57.7±4.21	**0.580**
Size (cm, mean ± SD)	6.33±3.10	5.96±0.47	4.6±0.86	**0.268**
Gender				**0.050**
Male	59(73.8%)	13(92.9%)	9(90%)	
Female	21(26.2%)	1(7.1%)	1(10%)	
Location				**0.002**
Cardia	33(41.2%)	11(78.6%)	1(10%)	
Non-cardia	47(58.8%)	3(21.4%)	9(90%)	
Grade				**0.823**
Poorly Differentiated	19(23.8%)	3(21.4%)	1(10%)	
Moderately Differentiated	50(62.5%)	9(64.3%)	7(70%)	
Well Differentiated	11(13.8%)	2(14.3%)	2(20%)	
Lymph node metastasis				**0.092**
Yes	67(83.8%)	12(85.7%)	6(60%)	
No	13(16.2%)	2(14.3%)	4(40%)	
Stage				**0.920**
I/II	19(23.8%)	3(21.4%)	2(20%)	
III/IV	61(76.2%)	11(78.6%)	8(80%)	
Depth of tumor invasion (T)				**0.292**
T2	20(25%)	2(14.3%)	1(10%)	
T3	43(53.8%)	8(57.1%)	5(50%)	
T4	17(21.2%)	4(28.6%)	4(40%)	
Type				**0.574**
Intestinal	55(68.8%)	11(78.6%)	6(60%)	
Diffuse	21(26.2%)	3(21.4%)	4(40%)	
Mixed	4(5%)	-	-	
*Helicobacter Pylori*				**0.759**
Positive	36(52.9%)	8(66.7%)	3(37.5%)	
Negative	32(47.1%)	4(33.3%)	5(62.5%)	

**Clinicopathological Features and KINDLIN1 Expression**

A significant correlation was observed between KINDLIN1 expression and gender , in which 92.9% (13/14) and 90% (9/10) of over-and underexpressed tumors, respectively, were observed among males (*P*=0.050). There was also a significant correlation between KINDLIN1 and tumor location, in which 11 out of 14 overexpressed tumors (78.6%) were located in the cardia. However, almost all of the KINDLIN1 underexpressed tumors (9/10, 90%) were located in the non-cardia. Therefore, the proximal tumors significantly showed higher level of KINDLIN1 mRNA expression compared to distal tumors (1.01±0.33 vs. -0.42±0.34 fold changes; *P*=0.002). A significant correlation was observed between KINDLIN1 expression and age among HP-positive cases (*P*=0.039). 

Although KINDLIN1 has no significant role in the grade of the tumor, the highest and lowest levels of KINDLIN1 expression were observed in poorly and moderately differentiated tumors, respectively (0.46±0.31 vs. 0.04±0.37 fold changes). KINDLIN1 overexpressed tumors interestingly presented with higher aggressive behavior, and most of such tumors showed lymph node involvement (12/14, 85.7%). However, KINDLIN1 underexpressed tumors demonstrated a lower tendency for lymph node metastasis (6/10, 60%). In KINDLIN1 overexpressed tumors, the tissues with primary tumor stages (I/II) showed lower expression levels than advanced tumor stages (III/IV; 2.32±0.15 vs. 3.21±0.38 fold changes). 

It was observed that there was a rising trend in the levels of KINDLIN1 mRNA expression toward the depth of invasion, in which T2 and T4 samples had the lowest and highest levels of expression, respectively (2.45±0.16 vs. 3.63±0.92 fold changes). We had HP results for only 68 cases of patients. Among HP-negative cases, tumors with metastatic lymph nodes had significantly higher levels of KINDLIN1 expression compared to non-metastatic tumors (0.53±034 vs. -2.87±1.08 fold changes; *P*=0.001).

## Discussion

KINDLIN1 is an essential regulator of cellular adhesion to extracellular matrix (ECM) proteins, which is dependent on focal adhesion kinase (FAK) (10). Regarding the regulatory role of integrin in cadherin-dependent cell adhesion, kindlin-1 might function as a hot spot among multiple signaling pathways during tumor progression. It has been shown that kindlin-1 triggers Epithelial-mesenchymal transition (EMT) via the downregulation of E-cadherin and upregulation of N-cadherin and fibronectin. KINDLIN1 over-expression has been reported in colon and lung tumor tissues (6). KINDLIN1 expression was significantly correlated with matrix invasion in colon cancer (11). 

Moreover, KINDLIN1 was correlated with larger tumor size and advanced TNM stage in hepatocellular carcinoma (HCC) (12). KINDLIN1 ectopic-expressed cells exhibit the activation of SMAD family member 2/3 (Smad-2/3) and several Smad-target genes such as Connective tissue growth factor (CTGF), Endothelin 1 (EDN1), and Matrix Metallopeptidase 9 (MMP9) (5). KINDLIN1 is an adaptor molecule that regulates the activation of transforming growth factor β (TGF-β)/Smad3 signaling through the integration of Type I TGFβ receptor (TβRI), Secretion Associated Ras Related GTPase 1A (SARA), and Smad3. KINDLIN1 and SARA regulate the phosphorylation of Smad3 in colorectal cancer. Although KINDLIN1 expression affected pancreatic cancer cell invasion, there was no effect on cell proliferation (13). The upregulation of KINDLIN1 stimulated the expression of EMT-related transcriptional factors, which are suppressors of Cadherin 1 (CDH1) and Zona occludens 1 (ZO-1) (9). 

**Fig. 2:**
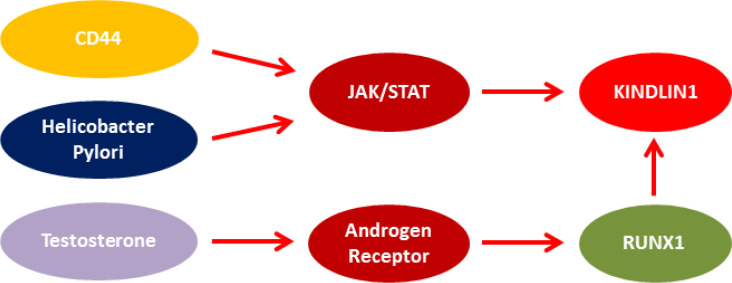
Probable correlation between KINDLIN1, sex, tumor location, and *Helicobacter Pylori*

Moreover, KINDLIN1 acts as an enhancer for the Leucine Rich Repeat Containing G Protein-Coupled Receptor 5 (LGR5) expression in colon cancer (14). A cohort study showed that KINDLIN1 overexpression was strongly correlated with metastasis-free survival of lung adenocarcinoma cases (5). KINDLIN1 and KINDLIN2 play opposite roles in the regulation of the EMT process of lung cancer. In non-small cell lung cancer (NSCLC), KINDLIN1 is mainly expressed in well-differentiated cells, leading to the inhibition of malignant progression, whereas KINDLIN2 expression is mostly observed in poorly differentiated cells as an inducer for the tumor progression and metastasis (15). We observed higher levels of KINDLIN1 expression in HP-positive tumors, which can be related to the role of HP in the activation of the Janus kinase (JAK)-signal transducer and activator of transcription (STAT) signaling pathway (16). The KINDLIN1 promoter sequence has several binding sites for STAT family members, such as STAT2-5. Therefore, HP can be involved indirectly in the upregulation of KINDLIN1 through the JAK/STAT signaling pathway (Figure 2). 

We found a significant correlation between KINDLIN1 expression and gender. It has been reported that androgen treatment induces the expression of runt-related transcription factor 1 (RUNX1) (17). RUNX1 is also the main transcription factor of KINDLIN1. It seems that the higher levels of KINDLIN1 expression in males compared with females can be related to the androgen-RUNX1 association. Therefore, in addition to the role of the androgen-RUNX1 association in prostate cancer, such pathway is also involved in GC progression among males. We observed that the anatomical structure of the stomach could be involved in KINDLIN1 expression, in which proximal tumors had higher levels of KINDLIN1 expression. It has been shown that there are different patterns of protein expression between cardia and non-cardia carcinoma in terms of several markers, such as CD44, Mucin 1 (MUC1), and Cyclin-Dependent Kinase Inhibitor 2A (CDKN2A). Cardia tumors had higher levels of CD44 compared to non-cardia tumors (18). CD44 activates JAK/STAT through the phosphorylation of STAT3 (19), leading to a probable upregulation of KINDLIN1 in cardia (Figure 2). 

## Conclusion

Regarding the prevalence of cardia gastric carcinoma among Iranians, it is really important to identify an efficient location-specific diagnostic marker. In the present study, we introduced KINDLIN1 as a location-specific marker for cardia gastric carcinoma. Since the levels of KINDLIN1 expression are higher in cardia tumors than non-cardia tumors, it can be considered a marker for the early detection of cardia tumors. However, the levels of KINDLIN1 in blood samples must be evaluated before it can be recommended as an effective non-invasive early detection marker of cardia tumors. Moreover, it was observed that KINDLIN1 could be used as a gender-dependent diagnostic marker of GC patients.

## Conflict of Interest

The authors declare that they have no conflicts of interest.

## Funding

This work was supported by a grant from the Vice Chancellor for Research at Mashhad University of Medical Sciences, No. 971292.
